# A "Sick House" for Private Nurses

**Published:** 1919-02-08

**Authors:** 


### A "Sick House" for Private Nurses.

*To the Editor of* The Hospital.

Sir,—As there is so much said and done just now to improve the standing and condition of nurses, may I be allowed to make a suggestion? There is a growing want, I am convinced, for, shall I call it, a "Sick House" for private nurses—at least, for nurses who live in hostels or rooms. These nurses, when they are slightly ill—for instance, a bronchial cold necessitating staying in bed or in one room—cannot get any or very little attention, and have to rely upon their friends to wait on them, and therefore cannot get well as quickly as they might. Often they wait until they are really ill before giving in.

Now the Florence Nightingale Hospital, Lisson Grove, N.W.1, meets the need of nurses who have acute non-infectious illnesses, or who require operations; for a small sum, 25s. weekly, they can get all they want, including doctors and nursing.

Could not a house be found and a club formed, so that any nurse could pay so much a year into the sick club, and then be taken into the "Sick House" for, say, 1s. a day? I believe if a house took in about 10 per cent. of the members and had a matron in charge and a doctor in attendance so many nurses would join that, once equipped, the house would be self-supporting. There would, of course, be rules as in all sick clubs, and there is no reason why it should be abused.

Matron.

